# Complex interrelationships among respiratory diseases and chronic multimorbidity: a longitudinal network analysis and implications for future viral respiratory pandemic preparedness

**DOI:** 10.3389/fepid.2025.1577333

**Published:** 2025-07-11

**Authors:** Daniel E. Zoughbie, Kyongsik Yun

**Affiliations:** ^1^ Institute of International Studies, University of California, Berkeley, Berkeley, CA, United States; ^2^ New England Institute of Complex Systems, Cambridge, MA, United States; ^3^ Computation and Neural Systems, California Institute of Technology, Pasadena, CA, United States

**Keywords:** network analysis, respiratory comorbidities, healthcare claims data, ICD-10 co-occurrence, chronic disease

## Abstract

**Introduction:**

Respiratory diseases such as asthma, chronic obstructive pulmonary disease (COPD), pneumonia, and acute respiratory failure contribute significantly to the global health burden, particularly when co-occurring with chronic systemic conditions. Understanding these interrelationships is essential for designing resilient and integrated healthcare systems, especially in the context of pandemic stress.

**Methods:**

We analyzed over 82 million de-identified healthcare claims from the Comprehensive Health Care Information System (CHIS), spanning 2020 to 2024. A disease co-occurrence matrix was constructed by identifying overlapping ICD-10 codes across individual patient timelines. Pairwise associations were quantified using Spearman's rank-order correlation. The resulting associations were visualized as an undirected disease network.

**Results:**

COPD (J44.9) and asthma (J45.909) emerged as central nodes in the multimorbidity network, showing strong associations with metabolic (E11.9-Type 2 diabetes, E78.5-hyperlipidemia), cardiovascular (I10-hypertension), and mental health disorders (F32.9-depression, F41.9-anxiety). A significant reduction in chronic disease management services was observed in 2022, corresponding with the peak impact of the COVID-19 pandemic, followed by a partial rebound in 2023.

**Discussion:**

The findings reveal the integrative role of respiratory diseases within broader patterns of multimorbidity, reinforcing the need for cross-disciplinary management approaches. The observed pandemic-related disruption in chronic care delivery highlights systemic vulnerabilities. Future preparedness strategies should integrate multimorbidity frameworks and ensure continuity of care for both respiratory and systemic conditions.

## Introduction

Respiratory diseases—including asthma, chronic obstructive pulmonary disease (COPD), pneumonia, and acute respiratory failure—account for a substantial proportion of the global disease burden (1). COPD alone ranks among the leading causes of death worldwide and significantly contributes to healthcare costs and lost productivity. Asthma, while often viewed as a less severe chronic condition, remains a major source of morbidity, particularly for individuals with limited healthcare access. Together, these respiratory disorders place considerable strain on health systems and require ongoing care, monitoring, and preventive strategies.

This challenge intensifies when respiratory diseases overlap with systemic comorbidities such as obesity, diabetes, cardiovascular disease, and mental health disorders (2, 3). Evidence increasingly points to shared inflammatory and metabolic pathways that exacerbate disease progression, elevating the likelihood of hospitalizations and poorer long-term outcomes. The COVID-19 pandemic, which disproportionately affected patients with multimorbidity, underscored the vulnerability of these populations (4). Efforts to maintain chronic care during pandemic surges revealed gaps in healthcare delivery models, particularly for those managing multiple chronic conditions.

Recognizing these challenges, the present study utilizes data from the Comprehensive Health Care Information System (CHIS) to explore the co-occurrence and interdependence of respiratory and systemic disorders. Through a robust network analysis of over 82 million healthcare claims, we identify key patterns of multimorbidity and illuminate potential pathways for targeted interventions. By highlighting how respiratory conditions integrate with other chronic diseases, this research aims to inform strategies that enhance resilience in healthcare systems, supporting both prevention and continuity of care during future viral respiratory pandemics.

## Methods

### Data sources

We used longitudinal patient-level data from the Comprehensive Health Care Information System (CHIS) for the state of New Hampshire, spanning January 1, 2020 to December 31, 2024. The dataset comprises 82,270,480 de-identified healthcare claims and includes encounter-level information such as ICD-10 diagnosis codes, patient demographics (age, sex, zip code), and healthcare utilization metrics (e.g., hospitalizations, outpatient visits).

The CHIS data is publicly available for qualified research purposes through the New Hampshire Insurance Department. Access requires a formal data request and approval. Detailed access procedures are available at: https://www.nh.gov/insurance/chis/.

### Variable definitions

We examined respiratory virus-related diagnoses identified using ICD-10 codes, including asthma (J45.909), chronic obstructive pulmonary disease or COPD (J44.9), acute respiratory failure (J96.01), pneumonia (J18.9), and COPD with acute exacerbation (J44.1). To identify frequent systemic comorbidities, we also analyzed co-occurring diagnoses for hypertension (I10), hyperlipidemia (E78.5), type 2 diabetes without complications (E11.9), gastroesophageal reflux disease (GERD, K21.9), and anxiety disorder (F41.9). Both primary and secondary diagnoses recorded during healthcare encounters were included in the analysis. Diagnoses were aggregated within 12-month windows to define co-occurrence at the patient level, ensuring that each disease pair was counted only once per patient per year.

The selection of ICD-10 codes was informed by prior clinical and epidemiological evidence highlighting common comorbidities in patients with respiratory conditions. Respiratory diseases such as asthma, COPD, pneumonia, and acute respiratory failure were chosen due to their strong associations with seasonal respiratory viruses and their high prevalence in public health surveillance. The systemic conditions included—such as hypertension, hyperlipidemia, and type 2 diabetes—represent common chronic diseases that are known to exacerbate respiratory illness severity and influence outcomes. GERD and anxiety were selected based on literature showing frequent co-occurrence with chronic respiratory conditions and their relevance in clinical symptom overlap and disease management. This targeted selection aimed to capture clinically meaningful and epidemiologically relevant comorbidity patterns.

### Statisticall and network analysis

We constructed a disease co-occurrence matrix based on pairwise frequencies of ICD-10 codes observed across individual patient timelines. The strength of association between each pair of diagnoses was assessed using Spearman's rank-order correlation. Statistical significance was defined at *p* < 0.05. Correlations that passed this threshold were used to build an undirected weighted network, where nodes represented ICD-10 diagnosis codes and edges represented significant correlations between diagnoses. The network was visualized using a force-directed layout to highlight clusters of diseases that frequently co-occurred. All analyses were conducted in Python 3.9 using the pandas library for data processing, scipy.stats for correlation calculations, statsmodels.stats.multitest for multiple testing correction, networkx for network construction, and matplotlib and Plotly for visualization.

### Ethics and data privacy

All records used in this study were fully de-identified before analysis in accordance with HIPAA privacy standards. The study protocol adhered to federal guidelines under 45 CFR 46 for the use of administrative health data. A data use agreement (DUA) was secured from the New Hampshire Insurance Department prior to data access. No individual-level informed consent was required. All analyses were conducted in compliance with both state-level data protection requirements and institutional policies governing research with de-identified data.

## Results

The study cohort comprised a total of 82,270,481 healthcare claims records from New Hampshire between 2020 and 2024. The mean age of individuals in the dataset was approximately 53.8 years (standard deviation: 21.7 years). The age distribution spanned from newborns (0 years) to a maximum of 90 years, with the interquartile range falling between 39 years (25th percentile) and 70 years (75th percentile). The median age was 58 years, indicating a middle-aged to older adult population, with a significant proportion over 65 years.

The gender distribution was as follows: female records totaled 47,231,052 (57.4%), male (M) records totaled 35,028,124 (42.6%), and a small number of records (11,305, which is 0.01%) were categorized as unspecified or other. This distribution reflects a moderate female predominance in healthcare utilization within the dataset.

Based on 2024 data, asthma commonly co-occurred with hyperlipidemia and anxiety disorders, while COPD frequently overlapped with hyperlipidemia and type-2 diabetes (Table 1). These patterns suggest consistent cardiometabolic and mental health comorbidities in patients with chronic respiratory conditions.

**Table 1:** Representative co-occurrences of respiratory and systemic conditions in 2024.

Condition 1	Condition 2	Co-occurrence count
Hyperlipidemia	COPD	10,084
Hyperlipidemia	Asthma	9,540
Anxiety disorder	Asthma	4,967
Type-2 diabetes	COPD	4,158
Type-2 diabetes	Asthma	4,575

**Figure 1:**
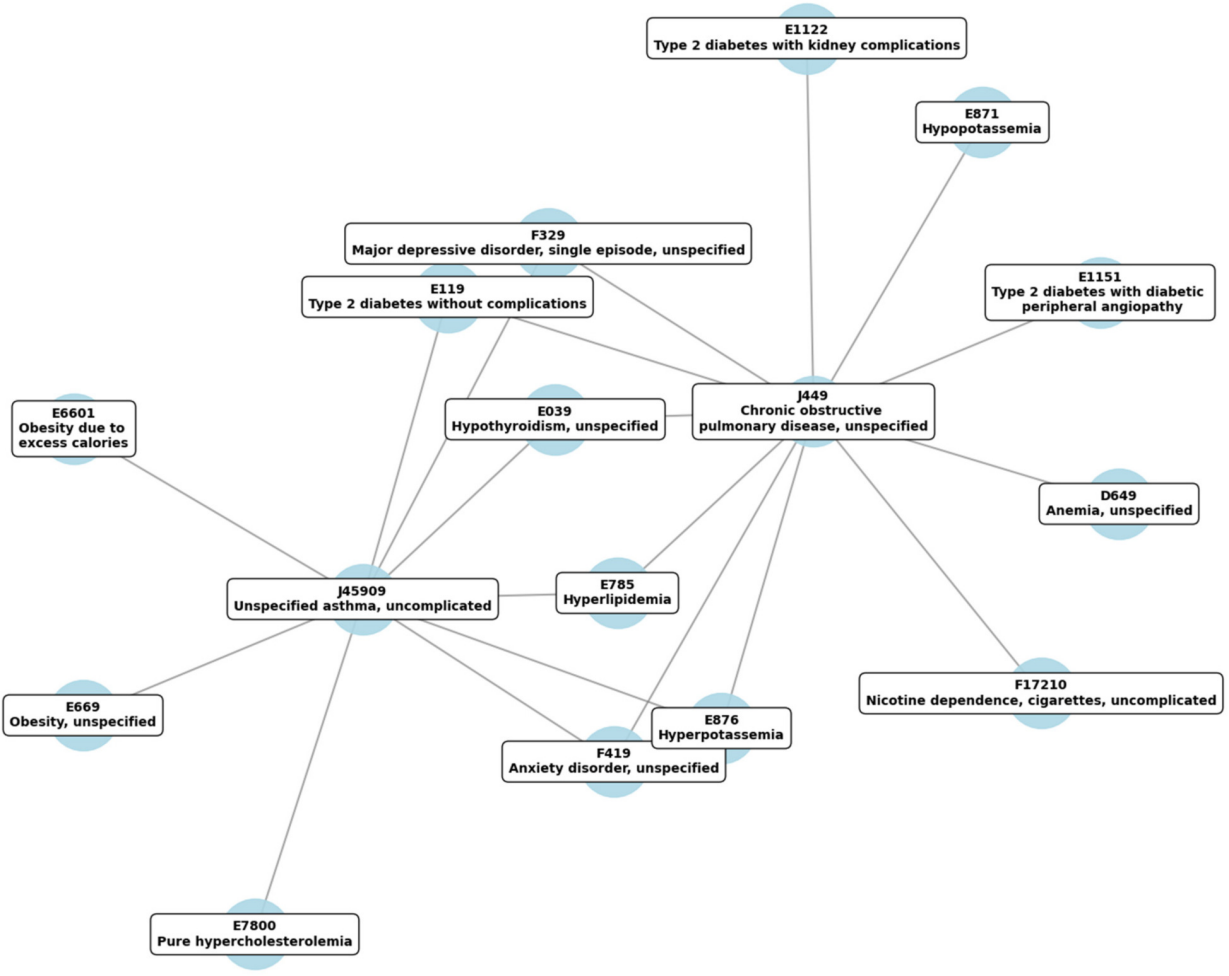
Temporal correlation network of highly comorbid diseases, based on ICD-10 codes, tracked from 2020 to 2024. Edges indicate significant temporal associations among conditions, with stronger connections signifying tighter comorbidity patterns.

Analysis of the longitudinal medical data from 2020 to 2024 revealed a tightly interconnected network of chronic diseases, with metabolic, respiratory, and mental health conditions at the center (Figure 1). Each node corresponds to a specific ICD-10 code, labeled with its clinical description, and edges signify the documented overlap of conditions within patient records.

Visual inspection of the network revealed two prominent respiratory nodes, J44.9 (COPD) and J45.909 (Asthma), each connecting to multiple systemic disorders. COPD (J44.9) showed strong associations with diabetes (E11.9, E11.22, E11.51), hyperlipidemia (E78.5), and mental health conditions (F41.9 for anxiety, F32.9 for depression). Similarly, Asthma (J45.909) was closely linked to metabolic disorders such as hyperlipidemia (E78.5), obesity (E66.01, E66.9), and electrolyte imbalances (E87.6 for hyperpotassemia), as well as anxiety (F41.9) and depression (F32.9).

Several metabolic codes formed secondary hubs, including hyperlipidemia (E78.5) and various diabetes-related diagnoses (E11.9, E11.22, E11.51). These nodes frequently connected back to J44.9 and J45.909, underscoring the recurring co-occurrence of metabolic abnormalities with chronic respiratory conditions. Mental health codes (F41.9 for anxiety and F32.9 for depressive disorder) also clustered around the primary respiratory nodes, highlighting the interplay between psychological well-being and chronic physical health conditions.

Overall, the network underscores the multifaceted nature of multimorbidity, wherein respiratory diseases coexist with metabolic, mental health, and other systemic disorders in a highly interconnected pattern. This visualization supports a broader understanding of how chronic respiratory conditions may integrate with or exacerbate comorbidities, reinforcing the value of comprehensive, interdisciplinary management strategies.

**Figure 2:**
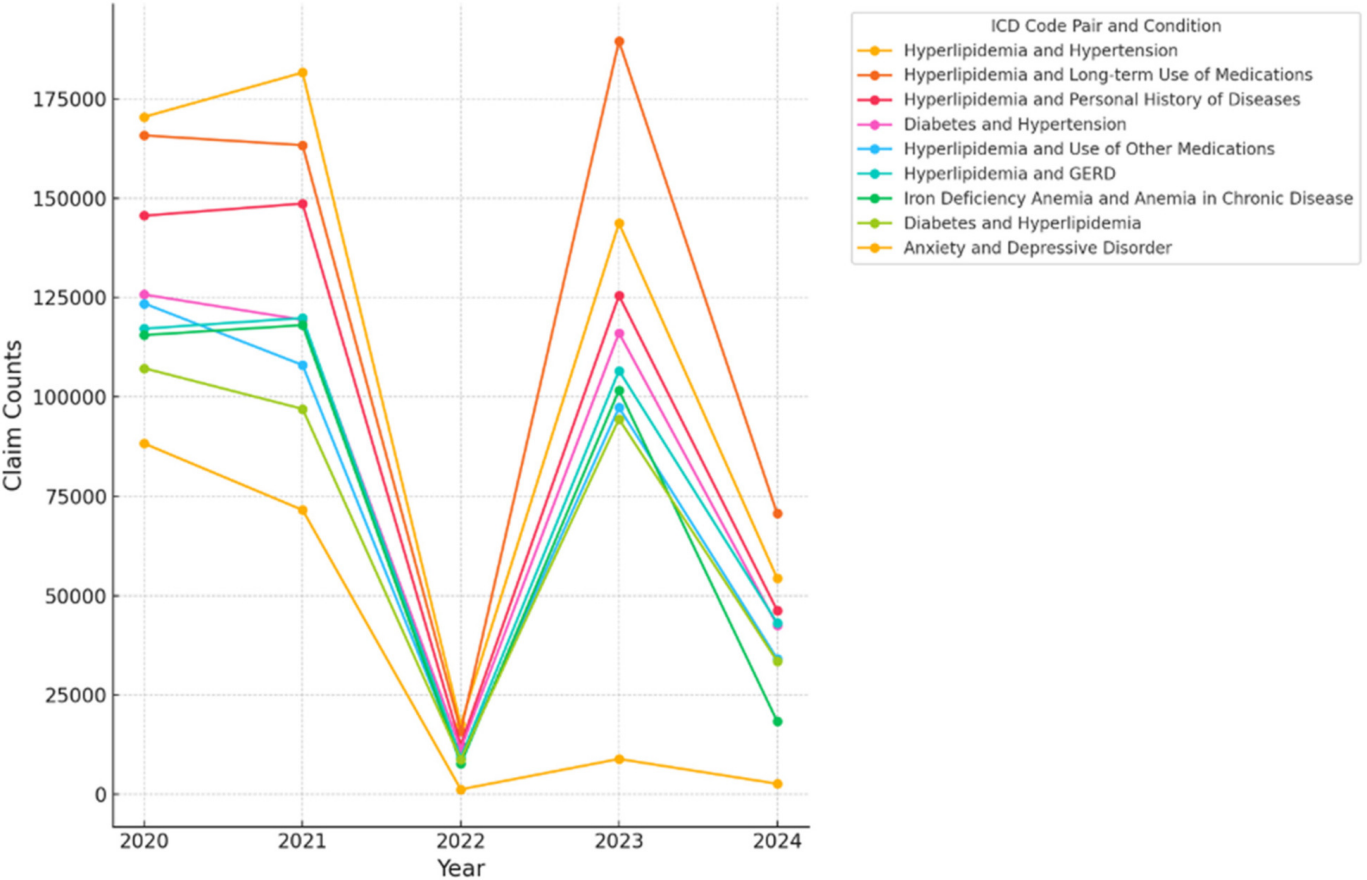
Longitudinal trends in Co-occurrence counts (2020–2024). The figure illustrates the longitudinal trends of co-occurrence counts for various ICD code pairs over the years, with counts for 2024 representing data up to June 2024. The ICD codes are labeled with their respective conditions: (‘E785’, ‘I10’)—Hyperlipidemia and Hypertension, (‘E785’, ‘Z79899’)—Hyperlipidemia and Long-term Use of Medications, (‘E785’, ‘Z87891’)—Hyperlipidemia and Personal History of Diseases, (‘E119’, ‘I10’)—Diabetes and Hypertension, (‘E785’, ‘Z7982’)—Hyperlipidemia and Use of Other Medications, (‘E785’, ‘K219’)—Hyperlipidemia and GERD, (‘D509’, ‘D631’)—Iron Deficiency Anemia and Anemia in Chronic Disease, (‘E119’, ‘E785’)—Diabetes and Hyperlipidemia, (‘F419’, ‘F329’)—Anxiety and Depressive Disorder.

Claim counts showed sharp declines in chronic disease management during 2022, coinciding with increased severity and complications in respiratory conditions. These trends reversed in 2023 as healthcare systems adapted (Figure 2; Supplementary Table S1).

## Discussion

This study highlights the complex interplay between respiratory diseases and chronic systemic conditions, underscoring the need for effective integrated care strategies to address multimorbidity. The findings reveal that inflammatory processes, metabolic dysfunction, and psychosocial stress act as compounding factors that worsen the progression of chronic respiratory conditions. Notably, metabolic abnormalities—particularly hyperglycemia—amplify pulmonary inflammation, exacerbating COPD and asthma (5). Concurrently, mental health disorders, including depression and anxiety, contribute to poorer self-care, reduced medication adherence, and increased healthcare utilization, perpetuating a cycle of disease burden (6).

The COVID-19 pandemic further exposed the vulnerability of multimorbid patients, as disruptions in routine care led to rapid deterioration in health status. A sharp decline in chronic disease management during 2022 coincided with worsening respiratory complications, illustrating how gaps in healthcare delivery can have cascading effects. This lapse underscores the urgent need for integrated care models that proactively coordinate services across specialties and leverage digital health technologies to bridge care gaps. Telehealth and remote patient monitoring have emerged as viable solutions for maintaining continuity of care, particularly for high-risk populations who require frequent monitoring but may face barriers to in-person services (4).

In response to these challenges, health systems should implement early detection and risk stratification strategies to identify high-risk patients with multiple comorbidities. Predictive analytics and electronic health record (EHR)-integrated screening tools could enable targeted interventions that prevent disease exacerbations, reduce hospitalizations, and improve long-term outcomes. Population-level screening programs focusing on patients with metabolic disorders, hypertension, or mental health conditions alongside respiratory diseases could facilitate more proactive healthcare interventions. Policies should also support the development of data-driven models to anticipate acute respiratory events, allowing for timely interventions such as vaccination campaigns, medication adherence programs, and lifestyle modifications.

Mental health emerges as a critical factor in respiratory disease management, given the strong co-occurrence between anxiety, depression, and chronic pulmonary conditions. Integrating mental health screening into routine care for patients with respiratory diseases could lead to earlier intervention and better overall disease management. Cognitive behavioral therapy, stress management techniques, and behavioral health referrals should be seamlessly incorporated into standard treatment plans. Addressing psychosocial determinants of health, including socioeconomic disparities and barriers to healthcare access, is equally vital in ensuring equitable health outcomes for multimorbid patients.

Beyond individual patient care, strengthening healthcare system resilience against future respiratory pandemics will require systemic investments in workforce training, interdisciplinary collaboration, and infrastructure. This study's lessons emphasize the importance of reinforcing supply chain security, ensuring hospital surge capacity, and developing flexible healthcare delivery models that adapt to shifting demands. By leveraging insights gained from longitudinal multimorbidity patterns, policymakers, and healthcare leaders can craft evidence-based strategies that enhance healthcare system responsiveness and sustainability in the face of emerging public health threats.

Ultimately, this longitudinal network analysis underscores that respiratory diseases do not exist in isolation but are deeply embedded within broader multimorbidity patterns. Recognizing and addressing these complex interrelationships through integrated, data-driven, and patient-centered approaches will be essential in reducing disease burden and fortifying healthcare systems against future respiratory pandemics. Future research should explore predictive modeling approaches to identify high-risk patient populations and optimize healthcare resource allocation, ultimately improving patient outcomes and system preparedness.

This study has several limitations. First, we did not apply multiple comparisons correction to the Spearman rank-order correlation analysis due to constraints in dataset structure and scale. As a result, the risk of false-positive associations among ICD-10 code pairs remains, and some reported correlations may not meet significance thresholds under more stringent statistical control. This limits the interpretability of marginal associations, especially when considering large numbers of disease pairs across multiple years. Second, inherent limitations in healthcare claims data—such as potential underreporting, miscoding of diagnoses, and variability in billing practices—may affect the accuracy of disease classification. In addition, differences in healthcare access, insurance coverage, and care-seeking behavior across demographic groups may introduce selection bias. These factors should be considered when interpreting disease co-occurrence patterns, and future studies using linked clinical records or validated registries may help address these issues.

## Data Availability

Publicly available datasets were analyzed in this study. This data can be found here: https://nhchis.com/.

## Supplementary material

Supplementary Table S1 Annual counts of co-occurring ICD-10 diagnosis code pairs involving respiratory conditions from 2020 to 2024 in New Hampshire CHIS data. This table highlights the temporal comorbidity between respiratory diseases—such as chronic obstructive pulmonary disease (J44.9), asthma (J45.909), and related conditions—and systemic or mental health disorders. Each ICD-10 pair is listed alongside yearly co-occurrence counts.
