# Supplementary material for: Complex interrelationships among respiratory diseases and chronic multimorbidity: a longitudinal network analysis and implications for future viral respiratory pandemic preparedness
Source: Front Epidemiol. 2025 Jul 11;5:1577333. doi: 10.3389/fepid.2025.1577333 (PMC12289556; doi:10.3389/fepid.2025.1577333)
Supplement: Supplementary Table S1 — Annual counts of co-occurring ICD-10 diagnosis code pairs involving respiratory conditions from 2020 to 2024 in New Hampshire CHIS data. This table highlights the temporal comorbidity between respiratory diseases—such as chronic obstructive pulmonary disease (J44.9), asthma (J45.909), and related conditions—and systemic or mental health disorders. Each ICD-10 pair is listed alongside yearly co-occurrence counts. [file Table1.docx]

**Supporting Materials**

Supporting Table 1. Annual counts of co-occurring ICD-10 diagnosis code pairs involving respiratory conditions from 2020 to 2024 in New Hampshire CHIS data. This table highlights the temporal comorbidity between respiratory diseases—such as chronic obstructive pulmonary disease (J44.9), asthma (J45.909), and related conditions—and systemic or mental health disorders. Each ICD-10 pair is listed alongside yearly co-occurrence counts.

| ICD_Code_Pair | Count_2020 | Count_2021 | Count_2022 | Count_2023 | Count_2024 |
| --- | --- | --- | --- | --- | --- |
| ('E785', 'J449') | 32003 | 30921 | 2324 | 25296 | 10084 |
| ('E785', 'J45909') | 24825 | 26568 | 2519 | 26316 | 9540 |
| ('F419', 'J45909') | 16823 | 15369 | 1543 | 13981 | 4967 |
| ('E119', 'J449') | 14509 | 15458 | 1195 | 13061 | 4158 |
| ('E119', 'J45909') | 12357 | 12819 | 929 | 12163 | 4575 |
| ('E039', 'J45909') | 11972 | 11334 | 1329 | 12895 | 5044 |
| ('E1122', 'J449') | 11051 | 9272 | 533 | 8697 | 2288 |
| ('F329', 'J45909') | 10165 | 9437 | 142 | 1035 | 482 |
| ('E039', 'J449') | 9084 | 8552 | 368 | 7464 | 2590 |
| ('F419', 'J449') | 8441 | 6426 | 324 | 5481 | 2713 |
| ('F17210', 'J449') | 8177 | 7547 | 723 | 6465 | 2886 |
| ('E6601', 'J45909') | 6595 | 5537 | 598 | 4500 | 1882 |
| ('E669', 'J45909') | 6328 | 7286 | 775 | 6215 | 2230 |
| ('F329', 'J449') | 6165 | 3606 | 40 | 490 | 132 |
| ('E876', 'J45909') | 5497 | 5328 | 334 | 3907 | 2364 |
| ('D649', 'J449') | 5482 | 4246 | 334 | 4129 | 1518 |
| ('E7800', 'J45909') | 5471 | 5686 | 535 | 6134 | 1682 |
| ('E1151', 'J449') | 5457 | 3954 | 237 | 2956 | 1100 |
| ('E871', 'J449') | 5421 | 4778 | 337 | 5517 | 1868 |
| ('E876', 'J449') | 5297 | 4822 | 265 | 4252 | 1875 |
